# Physical structure and absorption properties of tailor-made porous starch granules produced by selected amylolytic enzymes

**DOI:** 10.1371/journal.pone.0181372

**Published:** 2017-07-20

**Authors:** Yi-seul Jung, Byung-Hoo Lee, Sang-Ho Yoo

**Affiliations:** 1 Department of Food Science and Biotechnology, and Carbohydrate Bioproduct Research Center, Sejong University, Seoul, South Korea; 2 Department of Food Science & Biotechnology, College of BioNano Technology, Gachon University, Sungnam, South Korea; Universidad Nacional de Rosario, ARGENTINA

## Abstract

Porous starch granules (PSGs) with various pores and cavity sizes were prepared by amylolysis enzymes. The greatest hydrolysis rate on corn starch granule was observed with α-amylase, followed by gluco- and β-amylases. Temperature increase enhanced glucoamylase reaction rate more drastically than other enzyme treatments. Final hydrolysis level with glucoamylase reached to 66.9%, close to 67.5% of α-amylolysis. The α-amylase-treated PSGs displayed the greatest pore size and ratio of cavity-to-granule diameters. Gelatinization onset temperatures of PSGs increased to 72.1 (α-), 68.7 (β-), and 68.1°C (gluco-amylolysis) after 8 h; enthalpy changes of β- and gluco-amylase-treated PSGs increased to 13.4, and 13.1 J/g but α-amylase-treated one showed slightly reduced value of 8.5 J/g. Water holding capacities of PSGs were 209.7 (α-), 94.6 (β-), and 133.8% (gluco-amylolysis), and the untreated control had 89.1%; oil holding capacities of them showed 304.5, 182.7, and 211.5%, respectively, while the untreated control had 161.8%. Thus, enzyme types and their reaction conditions can be applied to generate desirable cavity and pore sizes in starch granules. This biocatalytic approach could contribute to develop tailor-made PSGs with distinct internal structure for specific uses in wide range of food, pharmaceutical and other industrial applications.

## Introduction

Biopolymers are widely used as encapsulated agents and loading-carrier materials in food, agricultural, and pharmaceutical area. Among all natural available biopolymers, starch is the most commonly applied in the biomaterial fields due to its beneficial advantages such as low-cost, low-toxicity, biodegradability, and renewability [1]. Despite these properties of starch materials, the industrial applications of starch are often limited because of its poor rheological properties and limited surface areas [2, 3]. Therefore, many studies have been undertaken to extend the starch application by using physical, chemical, and enzymatic methods to obtain desired specific properties and larger surface areas [4–6].

Porous starch has been interested because it has high surface area and accessible inner empty space. These properties can be applied in food, cosmetics, and other related industries as absorbents as well controlled delivery materials [7]. Porous structure of native and partially-gelatinized starch materials has been conventionally produced by physical and chemical modifications through extrusion, jet cooking, solvent exchanging, and sonication treatment [8–12]. Furthermore, porous starch granules (PSGs) can be produced by amylolytic enzymes without disintegration of granular integrity. When native starch granules are treated with amylolytic enzymes, semi-crystalline granular structure is degraded, leaving behind porous or broken starch granule structure [13].

For the enzymatic production of porous starch granule, α-, β-, and gluco-amylases, are the most common enzymes for structural modification of starch granule. α-Amylase can hydrolyze the internal α-1,4-glucosidic bonds of starch molecules with endo-hydrolytic activities except for those near the α-1,6 branching points. Basically, α-amylolysis occurs at any α-1,4-linkages in a random manner, resulting in wide pores on starch granules. β-Amylase is an exo-type enzyme that removes maltose units sequentially from non-reducing ends and stop the reaction near the α-1,6 branching points [14, 15]. Glucoamylase is another exo-acting enzyme that catalyzes the both α-1,4-and α-1,6-linkage hydrolyses starting from the non-reducing ends of the starch molecule chains and is able to drill sharp and deep channels on starch granules [16, 17]. Structures of the PSGs may allow certain chemicals to enter the central cavity of granules through the pores generated enzymatically. PSGs have protecting capability of labile compounds against light or oxygen exposure [3, 18]. Also, it was reported that PSGs prepared by the combined treatments of gluco- and α-amylases retained more coffee flavor compared to native starch [19].

Many researchers studied the formation of pores on starch granules by amylolytic enzymes and investigated application of PSGs products as encapsulation or carrier agent [3, 13, 20]. However, few attempts have been made regarding the preparation of granular starch with tailor-made pore size and its characteristics, especially of loading-carrier capacity of bioactive components. The optimal pore and cavity sizes of PSG may be different depending on the type and amount of targeted loading core materials. Also, the holding capacity in PSGs of loaded materials is another important parameter as an encapsulated or carrier agent. Thus, PSGs with tailor-made size of the pores and cavities by applying starch-active biocatalysts were prepared and their physicochemical properties and potentials as matrices for carrying lipophilic or hydrophilic bioactive chemicals were examined in this research.

## Materials and methods

### Materials and chemicals

Normal corn starch (NCS) was provided from Ingredion Inc. (Westchester, IL), and it was used as a granule starch to prepare PSGs. Three different types of starch hydrolyzing enzymes, glucoamylase from *Aspergillus niger* (Optidex L-400), β-amylase from barley (Optimalt^®^ BBA), and α-amylase from *Bacillus lichemiformis* (Spezyme Xtra), were obtained from the Dupont^™^ Genencor^®^ Science (Rochester, NY). Soybean oil was purchased from commercial products in a local market. Other reagent-grade chemicals were purchased from Sigma-Aldrich Chemical Co. (St. Louis, MO).

### Enzyme assay

One unit of hydrolyzing enzymes was determined as the amount of enzyme that catalyzes the production of 1 μmol of reducing sugars per minute. Individual enzyme assays were carried out in 50 mM sodium acetate buffer (optimal pH of each enzymes; glucoamylase: pH 4.5, α-amylase: pH 5.8, and β-amylase: pH 5.2) containing 1 mg/mL of soluble starch (Sigma-Aldrich Chemical Co., St. Louis, MO) at 40°C for 10 min. After the reaction, the amount of released reducing sugar was measured by the dinitrosalicylic acid (DNS) method, using glucose for glucoamylase activity or maltose as a standard for α- and β-amylases [21, 22].

### Preparation of PSGs by enzymatic treatment

Enzymatic reaction for producing the PSGs was carried out in 50 mM sodium acetate buffer (at optimal pHs of each enzyme) with 5% (w/v) of NCS. The individual hydrolyzing enzyme (800 U/g of starch) was added to the substrate mixture, and then the reaction was carried out at 30 or 40°C up to 8 h. After the reaction, 0.1 M NaOH was added to stop the enzyme activity. The dispersion was centrifuged (3,000 × *g*) at 4°C for 20 min, and the precipitated- modified starch pallet was washed twice with deionized water. The slurry was freeze-dried, and then the dried-sample was passed through a 100-mesh sieve. The sieved-product was stored in a desiccator at room temperature [18].

To determine the hydrolysis degree of PSGs, the diluted supernatant and DNS solution were mixed to ratio of 1:1. The mixture was kept in a boiling water bath for 5 min and was cooled in ice bath. The absorbance was measured at 575 nm using a UV/vis spectrophotometer (DU730, Beckman Coulter Inc., Brea, CA). The degree of hydrolysis was determined using the following equation:

$$Degree\;of\;hydrolysis=\left(V_{1}/V_{0}\right)\;\times \;\left(M/m\right)\;\times \;100;$$

where *V*_*0*_ is the weight of raw starch sample; *V*_*1*_ is the weight of total released reducing ends equivalent to glucose; *M* is the molar mass of glucose in starch (anhydroglucose unit, 162 g/mol); and *m* is the molar mass of d-glucose in supernatant after hydrolysis reaction (180 g/mol), respectively

### Field-emission scanning electron microscopy (FE-SEM)

The morphological properties of native corn starch and PSGs were observed by a field-emission scanning electron microscope (Supra 55 VP, Carl-Zeiss, Oberkochen, Germany). The samples were coated with platinum by a sputter coater (SCD 005, BAL-TEC, Walluf, Germany) and were observed at 15,000× magnification under an accelerating voltage of 3 kV. Surface pore diameters of starch granule were measured, and the diameters of the longest axes in each of 5 pores within an image for a total of 5 images per sample were quantified by using the ImageJ software (National Institutes of Health, Bethesda, MD).

### Confocal laser scanning microscopy (CLSM)

Merbromin-treated starch granules [23] were placed in immersion oil on a glass slide, overlaid with a glass cover slip, and observed with a TCS SP5 Confocal laser scanning microscopy (CLSM, Leica Microsystems, Mannheim, Germany) equipped with an argon laser. The excitation wavelength was 488 nm with 20% capacity and the light detected at the interval from 505 to 580 nm. During image acquisition, each line was scanned six times and averaged to reduce noise [24].

### Thermal properties of PSGs by differential scanning calorimeter

Thermal properties of PSGs were analyzed by using a differential scanning calorimeter (DSC-200, Netzsch, Selb, Germany). The starch sample (5 mg) was exactly weighed in an aluminum pan, and deionized water (15 mg) was added. This starch dispersion was equilibrated at ambient temperature for 24 h. The sealed sample was scanned from 20 to 120°C at a heating rate of 5°C/min, using an empty pan as a reference [25].

### Water holding capacity of PSGs

To measure the water holding capacity, starch (0.2 g, *dry basis*) was suspended in distilled water (4 mL) and shaken vigorously to fully disperse the starch. The suspended starch solution was allowed to stand for 10 min at ambient temperature. After the 10-min suspending period, the dispersion was centrifuged (1000 × *g*, 15 min), and the sediment was weighed. Water holding capacity of PSGs was calculated according to the following equation:

$$Water\;holding\;capacity\;\left(\%\right)=\left(W_{1}/W_{0}\right)\;\times \;100\text{;}$$

where *W*_*0*_ is the initial weight (g) of starch sample before treatment; *W*_*1*_ is the final weight (g) of starch sample after treatment. The analysis result was presented as the mean value of three replications [12].

### Oil holding capacity of PSGs

To measure the oil holding capacity, soybean oil (6 mL) was added to sample (0.5 g) in a graduated 50 mL centrifuge tube. The tube was vortexed for 1 min, left for 30 min and centrifuged for 25 min at 3000 × *g*. After 25 min the supernatant was removed, and the sediment was weighed. Oil holding capacity was calculated as:

$$Oil\;holding\;capacity\;\left(\%\right)=\left(W_{1}/W_{0}\right)\;\times \;100\text{;}$$

where *W*_*0*_ is the initial weight (g) of starch sample before treatment, and *W*_*1*_ is the is the final weight (g) of starch sample after treatment. The results presented are the mean values of three replications [26].

### Statistical analysis

All statistical analyses were performed by using IBM^®^ SPSS^®^ Statistics for Windows (Version 21.0, IBM Corporation, Armonk, NY). Statistical significance in the difference among the values was evaluated by Tukey’s test at *p* < 0.05.

## Results and discussion

### Effects of selected amylolytic enzymes on the hydrolysis pattern of native starch granule

**Fig 1:**
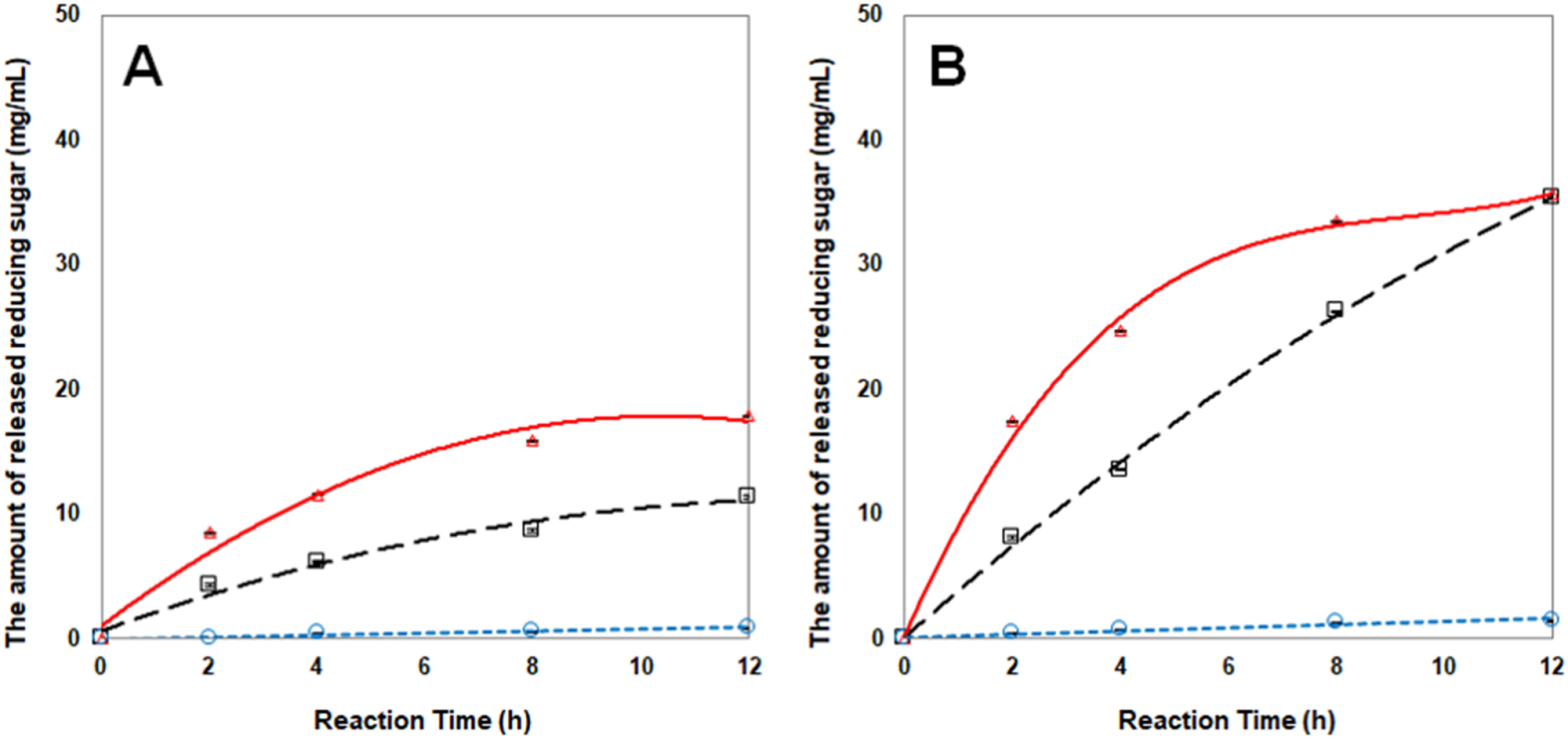
Biocatalytic hydrolysis pattern of normal corn starch granules treated with different amylolytic enzymes at 30°C (A) and 40°C (B). Solid line, α-amylase; short-dashed line, β-amylase; long-dashed line, glucoamylase.

The hydrolysis rates of NCS granules by the amylolytic enzymes were analyzed at two different temperatures, 30 and 40°C. The greatest hydrolysis rate of starch granule was observed when the NCS was hydrolyzed by α-amylase, and followed by glucoamylase and β-amylase, respectively (Table 1). Among three different amylases, the initial hydrolysis rates (*v*_0_) of α-amylase (5.8×10^−3^ s^-1^) on native starch granule was faster than glucoamylase (1.1×10^−3^ s^-1^) and β-amylase (7.8×10^−5^ s^-1^) at 40°C (Fig 1).

**Table 1:** Effects of reaction condition and enzyme type on hydrolysis degree of starch granules.

Reaction time (h)	Temperature (°C)	The type of enzymes applied^1)^		
		A	B	G
		Hydrolysis degree of native corn starch granule (%)		
2	30	16.10±0.16^bG^ ^2)^	0.08±0.02^dH^	8.24±0.27^cG^
	40	33.00±0.28^aD^	0.94±0.01^dD^	15.38±0.34^bE^
4	30	21.91±0.09^cF^	0.78±0.03^eG^	11.67±0.14^dF^
	40	46.71±0.52^aC^	1.33±0.01^eC^	25.64±0.04^bC^
8	30	30.19±0.07^cE^	1.09±0.01^fF^	16.44±0.16^dE^
	40	63.48±0.56^aB^	2.40±0.03^eB^	49.67±0.42^bB^
12	30	33.80±0.09^bD^	1.70±0.03^dE^	21.57±0.24^cD^
	40	67.46±0.28^aA^	2.81±0.01^dA^	66.88±0.67^aA^

^1)^ A, α-amylase; B, β-amylase; G, glucoamylase.

^2)^ Means of four replicates. The values with different letters of superscript within the same column (A-H) and row (a-f) are significantly different at *p*<0.05.

Thus, it was clearly observed that endo-activity of α-amylase can hydrolyze the starch granules more effectively than exo-types of glucoamylase and β-amylase. The total amount of released reducing sugars by α-amylase reaction increased as much as twice throughout whole reaction time period up to 12 h by increasing reaction temperature from 30 to 40°C. The degree of hydrolysis at 30°C for 12 h (33.8%) was similar to the resulted at 40°C within 2 h (33.0%) by α-amylase treatment.

The temperature effect on the hydrolysis level of glucoamylase was even greater, thus it produced more than three times larger amount of reducing sugars at 8 h and thereafter. As a result, the increase of the reaction temperature by 10°C enhanced the *v*_0_ of glucoamylase more drastically than other two enzymes, and eventually the degree of hydrolysis by glucoamylase reached the plateau (66.88%) of which the level was very close to that of α-amylase (67.46%) after 12 h. The amount of released glucose after 4-h α-amylase treatment was similar to that of 8-h glucoamylase treatment at 40°C. It was caused by the difference in reaction pattern between α-amylase and glucoamylase. α-Amylase is an endo-type enzyme that can randomly hydrolyzed α-1,4 glycosidic bonds of starch, and glucoamylase is an exo-type enzyme that can not only hydrolyze α-1,4 glycosidic bonds but also α-1,6 glycosidic bonds [27]. Thus, glucoamylase and α-amylase reached the same degree of hydrolysis under the extended reaction time period, even though the *v*_0_ of α-amylase was faster.

β-Amylolysis for 12 h showed that the starch granules were hydrolyzed in 1.7 and 2.8% of degrees at 30 and 40°C, respectively. Compared to these α-amylase and glucoamylase enzymes, β-amylase hydrolyzed the starch granules very slowly, and it was unable to destruct native starch granule effectively. Also, it was well reported that β-amylase hydrolyzed very slowly on all the starches over α-amylase [28]. Although both glucoamylase and β-amylase are exo-hydrolytic enzymes, glucoamylase can uniquely hydrolyze α-1,6 branched points and the action of this enzyme resulted in incomparably higher degree of hydrolysis than β-amylase reaction.

### Morphological properties of amylase-treated starch granules

**Fig 2:**
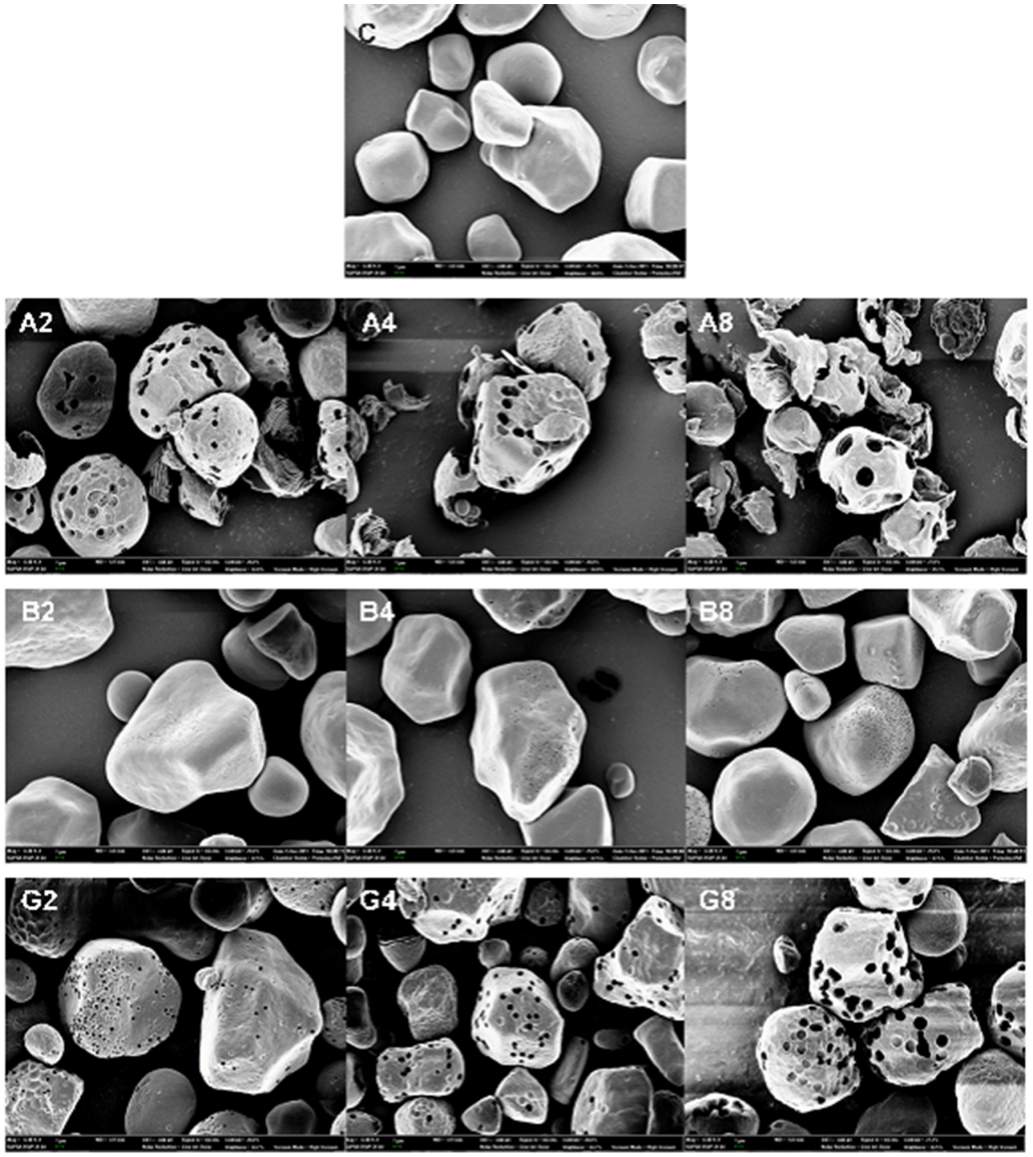
FE-SEM images of porous starch granules produced by enzymatic hydrolysis at 40°C. C, control (NCS); A, α-amylase; B, β-amylase; G, glucoamylase. The numbers in the image panels mean the reaction time in hour scale. For example, A2 means that native starch granule was treated with α-amylase for 2 h. The magnification is 6,500 ×.

In the FE-SEM analysis, α-amylase-treated starch granules showed relatively fewer number of large pores with many cracks whereas glucoamylase-treated ones had lots of small pores under the same conditions (Fig 2). The size and number of surface pores on starch granules produced by starch hydrolyzing enzymes were presented at different reaction time and temperature in Tables 2 and 3. The range of pore diameters generated by α-amylase was more diverse and wider (600–1,200 nm) compared to that of glucoamylase (500–600 nm). The β-amylase-treated granules at 40°C had the large number of nano-size pores, of which diameters were less than 100 nm on the surface. The number of pores on the surface of α-amylase-treated granules decreased along with the hydrolysis time, because small pores produced initially became larger and then merged into bigger size of pores. As the reaction time and temperature increased, the average size of pores became larger by α-amylolysis and the number of broken PSGs with the pore diameter of over 1 μm were increased. Between two exo-type enzymes, only glucoamylase hydrolyzes 1,6-linkage, which can facilitate to enhance amylolytic capability toward raw starch granule. The other exo-type β-amylase consistently showed low hydrolytic efficiency on the starch granule, thus this enzymatic reaction never reached to the hydrolysis level of other two enzymes. Thus, the discrepancy in the reaction patterns on starch granule among these amylolytic enzymes were considered as the crucial reason to produce different patterns of surface pores.

**Table 2:** Effects of reaction condition and enzyme type on pore size distribution of the starch granule surface.

Reaction time (h)	Temperature (°C)	The type of enzymes applied^1)^		
		A	B	G
		Surface pore size distribution in diameters (nm)		
2	30	100–300	<100	150–200
	40	500–900	<100	200–400
4	30	200–500	<100	300–400
	40	>1,200	<120	500–800
8	30	600–1,200	<100	500–600
	40	>1,800	<150	>1,000

^1)^ A, α-amylase; B, β-amylase; and G, glucoamylase.

**Table 3:** Effects of reaction condition and enzyme type on the number of surface pores.

Reaction time (h)	Temperature (°C)	The type of enzymes applied^1)^					
		A		B		G	
		The number of granule surface pores					
2	30	80–100	(-)^2)^	4–10	(-)	20–50	(-)
	40	30–50	(+)	30–80	(-)	50–70	(-)
4	30	40–60	(-)	4–15	(-)	30–80	(-)
	40	<20	(++)	100–160	(-)	40–70	(+)
8	30	30–50	(+)	10–20	(-)	50–80	(-)
	40	<10	(+++)	140–200	(-)	20–30	(++)

^1)^ A, α-amylase; B, β-amylase; and G, glucoamylase.

^2)^ The signs in the parenthesis indicate disintegration degree of starch granule in terms of the number of broken granules (-, none; +, a few; ++, often; +++, almost).

When the same degrees of starch hydrolysis were obtained from an enzyme treatment with different reaction temperatures and times, it was noticed that the size and morphology of pores on surface of starch granules were almost identical. Thus, amylolysis at 40°C for 2–4 h would be better applicable than the treatment at 30°C for 8 h. In this observation, it was affirmatively supported by the previous research [29] that the size of pores on starch granule could be manipulated by selecting proper type of amylase and by controlling its reaction condition

### Internal structures of PSGs analyzed by confocal laser scanning microscope

**Fig 3:**
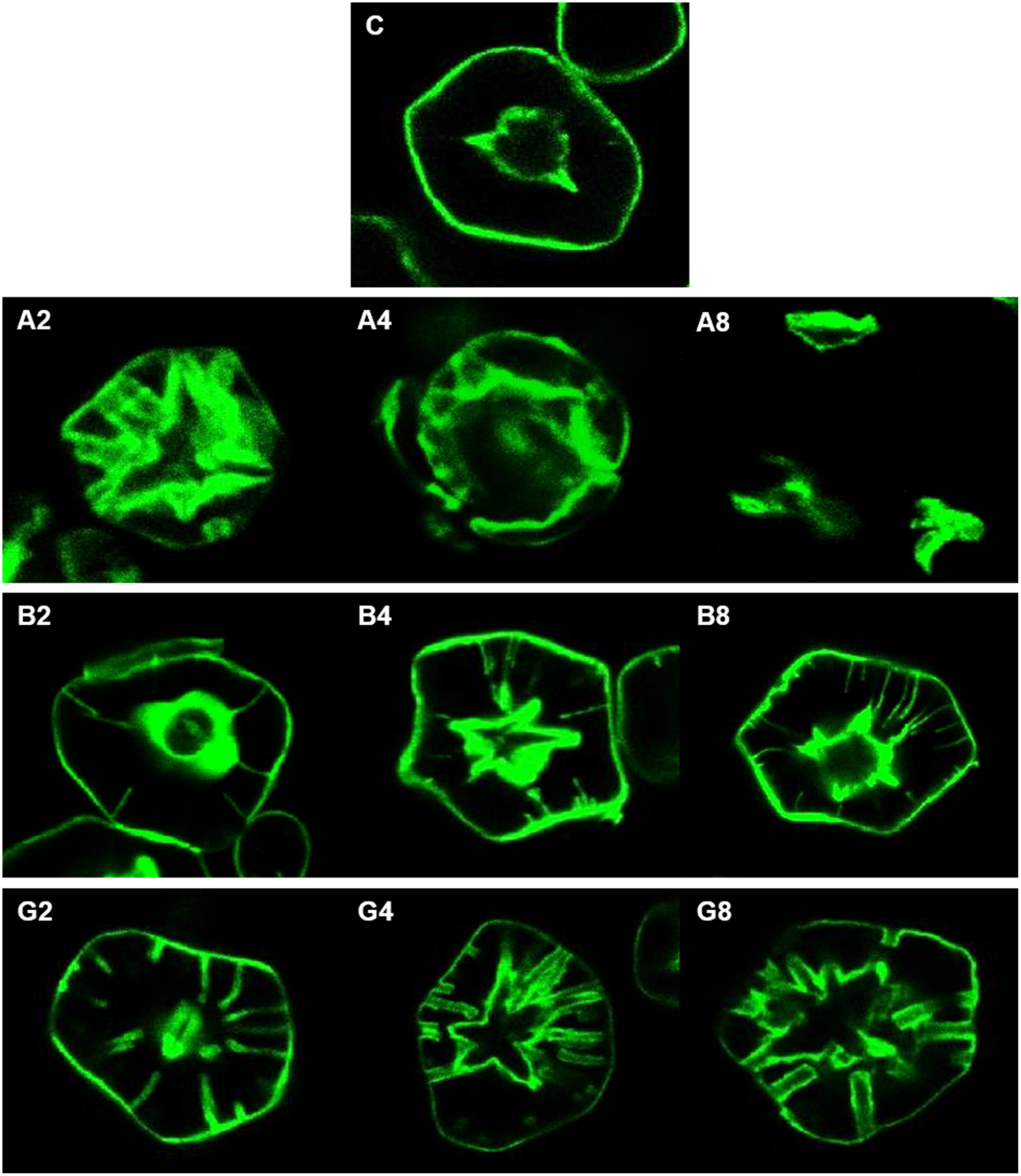
CLSM images for control and hydrolyzed starches after hydrolysis at 40°C for different time. C, control (NCS); A, α-amylase; B, β-amylase; and G, glucoamylase. The number means the reaction time. For example, A2 means that α-amylase was treated on starch granule for 2 h.

The internal structure of enzyme-treated starch granules at 40°C during different reaction time internals was visualized by means of the confocal microscopic method (Fig 3). When starch granules were treated with α-amylase, internal cavity in the hilum and surface pore were enlarged simultaneously. After 8-h reaction of α-amylase at 40°C, the large proportion of PSGs were broken apart by excessive hydrolysis. It can be reasonably speculated that the endo-active α-amylase accessed to the internal structure of PSGs and then rather freely hydrolyzed amorphous and imperfect crystalline regions inside. Thus the enlarging rate of internal cavity and surface pores was relatively fast, and the broken particles were easily observed due to excessively expanded cavity and pores. Shrestha, Blazek [30] reported that by ‘inside out’ digestion, the surface pores merged together forming larger channels and resulting in hollow interiors. The β-amylase-treated PSGs showed unchanged size of central cavity inside compared to untreated corn starch granule and formed only deep and narrow pores, while the channel from surface to internal cavity became expand through continuous enzyme reaction. When glucoamylase was treated on starch granules, the cavity and pores became wider and pores were being connected to cavity along with reaction time. However, the enlarging rate of cavity and pore sizes never reached to the degrees obtained with α-amylase. As mentioned earlier, glucoamylase displayed lower hydrolysis rate than α-amylase, but made wide cavity, unlike β-amylase, resulting from the action of glucoamylase which can not only hydrolyze α-1,4 glycosidic bonds, but also α-1,6 glycosidic bonds.

The ratio of internal cavity diameter (D_c_) over granule diameter (D_g_) of PSGs produced by amylolytic enzymes at 40°C was determined from different reaction time points (Table 4). After 2 h of α-amylase treatment, this D_c_/D_g_ ratio increased to 0.54, which was 57% greater than 0.31 of untreated NCS. Meanwhile, the D_c_/D_g_ ratio after β-amylase treatment kept constant to 0.37, which was very similar with that of NCS. Because β-amylase had very low hydrolysis efficiency on raw starch granules, it was expected that internal cavity may not be significantly enlarged. In case of another exo-type glucoamylase that harbors α-1,6 linkage hydrolytic activity, the ratio of D_c_/D_g_ was 0.52 after 8-h incubation at 40°C, which was equivalent to the value of the PSGs treated with α-amylase for 2 h implying more efficient hydrolysis of starch granule than did β-amylase.

**Table 4:** Effect of amylolytic enzymes on central cavity size ratio over granule diameters in the porous starch granules.

Reaction Time (h)	The type of enzymes applied^1)^		
	A^2)^	B	G
	D_c_/D_g_^3)^		
0	0.31±0.01^d)^ ^4)^		
2	0.54±0.02^b^	0.037±0.01^cd^	0.38±0.02^c^
4	0.66±0.03^a^	0.36±0.02^cd^	0.40±0.02^c^
8	nd^5)^	0.38±0.02^c^	0.52±0.03^b^

^1)^ Enzymatic reaction was carried out at 40°C for different time intervals with 40 U/mL of amylase activity, and initial starch concentration was 5% (w/v).

^2)^ A, α-amylase; B, β-amylase; and G, glucoamylase.

^3)^ The D_c_/D_g_ means that the ratio of surface pore to whole granule diameters of porous corn starch granules.

^4)^ Means of four replicates. The values with different letters of superscript (a-d) are significantly different at *p*<0.05.

^5)^ nd, not determined.

### Thermal transition properties of PSGs

Gelatinization temperature (T_gel_) of starch granule in the presence of water has been known to be affected by the structure of crystalline region, which corresponds to the external chain of amylopectin molecules formed with the double helical structure [31, 32]. Compared to the gelatinization onset temperature (T_o_ = 66.7°C) and enthalpy change (*Δ*H_gel_ = 10.6 J/g) of NCS, enzymatic digestion of native starch granule caused to increase T_o_ by at least 0.7°C from 2-h gluco-amylolysis and up to 5.3°C from 8-h α-amylolysis (Table 5). Previous research showed that corn starch granules treated with amylolytic enzymes displayed higher gelatinization temperature [3]. By α-amylase hydrolysis, it was also reported that the significant increases in T_o_ and T_p_ were observed from waxy rice and barley starch, respectively [33, 34].

**Table 5:** Melting properties of crystalline structure in the PSGs.

PSG sample^1)^	Thermal transition parameters^2)^				
	T_o_	T_p_	T_c_	*Δ*T	*Δ*H_gel_
	(°C)				(J/g)
NCS	66.7±0.1^h^ ^3)^	70.7±0.1^g^	76.3±0.6^e^	9.6±0.4^b^	10.6±0.3^b^
A2^4)^	70.3±0.1^c^	74.9±0.1^b^	81.8±0.1^a^	11.5±0.0^a^	10.9±0.3^b^
A4	71.3±0.1^b^	75.9±0.1^a^	82.4±0.0^a^	11.2±0.1^a^	9.2±0.4^c^
A8	72.1±0.1^a^	76.3±0.1^a^	82.7±0.1^a^	10.6±0.1^a^	8.5±0.0^d^
B2	69.5±0.2^d^	74.0±0.1^c^	80.4±0.1^b^	10.8±0.4^a^	13.3±0.6^a^
B4	68.9±0.1^e^	73.4±0.1^d^	80.0±0.1^b^	11.2±0.^a^	13.1±0.2^a^
B8	68.7±0.1^e^	73.2±0.3^d^	79.6±0.4^bc^	11.1±0.3^a^	13.4±0.2^a^
G2	67.4±0.1^g^	71.8±0.1^f^	78.3±0.1^d^	10.9±0.2^a^	13.2±0.4^a^
G4	67.5±0.0^g^	71.9±0.1^ef^	78.2±0.2^d^	10.7±0.2^a^	13.4±0.4^a^
G8	68.1±0.1^f^	72.3±0.0^e^	78.7±0.4^cd^	10.6±0.4^a^	13.1±0.2^a^

^1)^ Enzymatic reaction was carried out at 40°C for different time intervals with 40 U/mL of amylase activity, and initial starch concentration was 5% (w/v).

^2)^ To, Tp, Tc, ΔT, and ΔHgel represent the onset, peak, conclusion, the gelatinization temperature range and enthalpy of gelatinization, respectively; ΔT = Tc-To.

^3)^ Means of three replicates. The values in the same column with different letters of superscript (a-h) are significantly different at p<0.05.

^4)^ A, α-amylase; B, β-amylase; G, glucoamylase. The number means reaction time. For example, A2 means that α-amylase treated for 2 h.

From our enzymatic hydrolysis study, it was shown that endo-type α-amylase increased the T_o_ of starch granule gelatinization much greater, then followed by the other two exo-type β-amylase and glucoamylase, respectively. Slight increase in the T_gel_ range (*Δ*T; T_c_-T_o_) of starch granules was also observed by all the enzyme treatments in this melting phenomenon but there was no significant difference among the enzyme-treated samples. The endo-type enzyme might be much easier to access and hydrolyze both amorphous and imperfect crystalline regions inside starch granule in the diffusive mode, once the enzyme made complete holes in the centripetal mode [35]. As a result, this endo-amylolysis led to slow but steady decrease in *Δ*H_gel_ over the reaction time [33]. Because exo-type amylolytic enzymes preferentially degraded the amorphous regions mostly from outer layers of starch granule without attacking weak crystalline region, the increase in proportion of crystalline region was expected from the PSGs produced exo-catalytically [36], and thus resulted in significant increases in *Δ*H_gel_ within initial 2 h of exo-amylolysis.

### Water and oil holding capacity of PSGs

In an effort to utilize the porous structure of starch granule as a delivery system of nutrients or drugs, its loading capacity was evaluated by applying two different liquids, water and oil. When enzymatic hydrolysis was pursued at 40°C, the reaction time influenced on water holding capacity (WHC) of starch granules (Table 6). As the enzymatic reaction time increased, the WHC of α-amylase-treated PSGs increased from 148.9 to 225.6% at 4 h, then decreased to 209.7% after 8 h. As a control, the enzyme-untreated NCS granule displayed 89.0% of WHC. This value of glucoamylase-treated PSGs increased steadily to 133.8% after 8 h while β-amylase treatment on starch granule did not change it significantly up to 8-h reaction. It was previously shown that the starch granules structure was disintegrated and led to decrease in the holding capacity of water by excess treatment of enzymes [3], which was in good agreement with our result that the WHC decreased in 8-h α-amylase-treated PSGs.

**Table 6:** Water and oil holding capacity of PSGs.

PSG sample^1)^	Water holding capacity (%)	Oil holding capacity (%)
NCS	89.0±0.7^h^ ^2)^	161.8±1.1^h^
A2^3)^	148.9±7.0^c^	233.1±1.0^c^
A4	225.7±0.7^a^	286.2±1.7^b^
A8	209.7±0.4^b^	304.5±1.4^a^
B2	100.3±0.7^f^	177.6±2.3^f^
B4	102.3±0.4^f^	180.5±0.5^g^
B8	94.6±0.7^g^	182.7±1.2^fg^
G2	117.4±7.0^e^	185.5±2.4^f^
G4	125.6±0.7^de^	190.8±1.4^e^
G8	133.8±2.5^d^	211.5±1.3^d^

^1)^ Enzymatic reaction was carried out at 40°C for different time intervals with 40 U/mL of amylase activity, and initial starch concentration was 5% (w/v).

^2)^ Means of three replicates. The values in the same column (a-h) with superscripts are significantly different at *p*<0.05.

^3)^ A, α-amylase; B, β-amylase; G, glucoamylase. The number means reaction time. For example, A2 means that α-amylase treated for 2 h.

Oil holding capacity (OHC) of starch granules was also affected by enzymatic hydrolysis at 40°C (Table 6). As a control, the OHC of NCS was determined to be 161.8%. As the reaction time increased, the OHC of α-amylase- and glucoamylase-treated PSGs increased from 233.1 and 185.5% to 304.5 and 211.5%, respectively, whereas the OHC of β-amylase-treated PSGs increased slightly at 2 h but did not show any significant change thereafter. Previous study showed that that the maximum holding capacity of PSGs did not appear at the maximum hydrolysis due to the decrease in surface area resulting from an increase in pore size [37]. Furthermore, they also reported that water and oil holding capacity decreased as the pore size was over-enlarged.

## Conclusion

In this study, different types of PSGs were prepared by amylolytic enzymes such as α-amylase, β-amylase and glucoamylase which were able to generate various pores and cavity sizes on the surface. Unique structural and biochemical properties of PSGs can be designed and produced by different amylolytic enzyme treatments. When we utilized the internal cavity and expended surface area of PSGs, generated by these enzyme treatments, the holding capacity of water and oil was significantly increased with optimal porous structure production. Thus, different types of α-amylolytic enzymes and their various reaction conditions can be applied to produce desirable cavity and pore sizes in raw starch granules. This biocatalytic approach could apply to design tailor-made PSGs with distinct internal structure for specific uses as a novel carbohydrate-based delivery system or encapsulant in wide range of food, pharmaceutical and other industrial applications.
